# Supplementary material for: Language experience in LSF development: Behavioral evidence from a sentence repetition task
Source: PLoS One. 2020 Nov 17;15(11):e0236729. doi: 10.1371/journal.pone.0236729 (PMC7671551; doi:10.1371/journal.pone.0236729)
Supplement: S1 Table — SASS. Size and Shape Specifiers. (DOCX) [file pone.0236729.s005.docx]

**S1 Table. All sentences. SASS. Size and Shape Specifiers**

| **Item** | **Sign Span** | **Syntactic Complexity** | **Sentence content and inflections**  **GLOSS & *approximate translation*** |
| --- | --- | --- | --- |
| 1 | 2 | Easy | DAD – WAIT |
|  |  |  | *Daddy is waiting* |
| 2 | 2 | Easy | RUN – FORBIDDEN |
|  |  |  | *It is forbidden to run* |
| 3 | 3 | Easy | FRIENDS – MEET – KISS |
|  |  |  | *Friends meet and kiss each other* |
| 4 | 3 | Intermediate easy | GLASS– CL: glasses are piled up –FALTER |
|  |  |  | *I pick up the glasses and stack them, and the pile wobbles* |
| 5 | 2 | Easy | TOGETHER – RUN |
|  |  |  | *We run together* |
| 6 | 4 | Intermediate easy | MAN – CL: C handshape GLASS for drink action – TASTE – DISGUSTING |
|  |  |  | *A man is disgusted with what he drinks* |
| 7 | 4 | Intermediate easy | BONE – SMALL (SASS) – DOG – DISAPPOINTED |
|  |  |  | *The dog is disappointed because its bone is small* |
| 8 | 4 | Intermediate difficult | CAT – CL: cat – DOG – CL: dog – CL: cat FOLLOW |
|  |  |  | *The dog is following the cat* |
| 9 | 5 | Intermediate difficult | CHILD – FRENCH FRIES – CL: eat French fries – TASTE – DELICIOUS |
|  |  |  | *The child eats French fries and they’re delicious* |
| 10 | 3 | Intermediate easy | TEACHER – BOOK – CL: the teacher distributes books |
|  |  |  | *The teacher distributes the books* |
| 11 | 5 | Intermediate easy | DAD – MUM – CL: parents lie down – CHILD – CL: slip between parents |
|  |  |  | *The parents are lying in their bed and the child slips in the middle* |
| 12 | 5 | Intermediate difficult | CHILD – CL: slide down the slide – CL: go back to the slide – CL: slide down – CL: go back |
|  |  |  | *The child slides down the slide, goes back to the slide and slides down once again* |
| 13 | 5 | Intermediate difficult | CAKE – THREE – CL: big cake – CL: medium cake – CL: small cake |
|  |  |  | *There are three cakes: a big one, a medium one and a small one* |
| 14 | 5 | Intermediate difficult | CHILDREN – HAT – CL: hat on the head – CL: put the hat on the child’s head – CL: match the hat to the child’s head |
|  |  |  | *I take the hat that I have on my head, I put it on the child’s head and I fit it to the child’s head.* |
| 15 | 5 | Complex | BATHROOM – BATHTUB – CHILD + CL: bathtub – CL: the child gets in the tub + CL: bathtub – WASH |
|  |  |  | *In the bathroom, the child gets in the tub, and soaps himself* |
| 16 | 5 | Complex | BOX – CANDY + CL: box – EAT + CL: box – NO CANDY LEFT + CL: box – DISAPPOINTED + CL: box + CL: no candy left |
|  |  |  | *I ate all the candies that were in the box and there’s nothing left, so I am disappointed* |
| 17 | 4 | Intermediate easy | MAN – CL: drink – CL: glass fall – CL: flowing liquid |
|  |  |  | *A man drinks, drops his glass and spills water all over himself* |
| 18 | 5 | Complex | EAT – FINISHED – GO UPSTAIRS – TV – CL: watch TV |
|  |  |  | *As soon as I have finished eating, I go upstairs and I watch TV* |
| 19 | 5 | Complex | GRANDFATHER – BOOK – TELL – CHILDREN – CL: children listen the story |
|  |  |  | *The grandfather tells a story to his grandchildren and the children listen to him carefully* |
| 20 | 6 | Complex | BEFORE – CHRISTMAS – GIFT – NONE – NOW – A LOT |
|  |  |  | *Before, we offered few gifts at Christmas; now there are a lot* |
