# Supplementary figures and images for: Language experience in LSF development: Behavioral evidence from a sentence repetition task
Source: PLoS One. 2020 Nov 17;15(11):e0236729. doi: 10.1371/journal.pone.0236729 (PMC7671551; doi:10.1371/journal.pone.0236729)

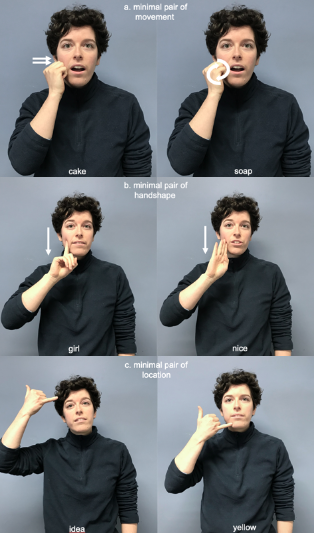

Supplement: S1 Fig — Minimal pairs in LSF in which changing the internal movement (a. CAKE/SOAP), the handshape (b. GIRL/NICE) or the location (c. IDEA/YELLOW) changes the meaning. (TIFF) [file pone.0236729.s006.tiff]

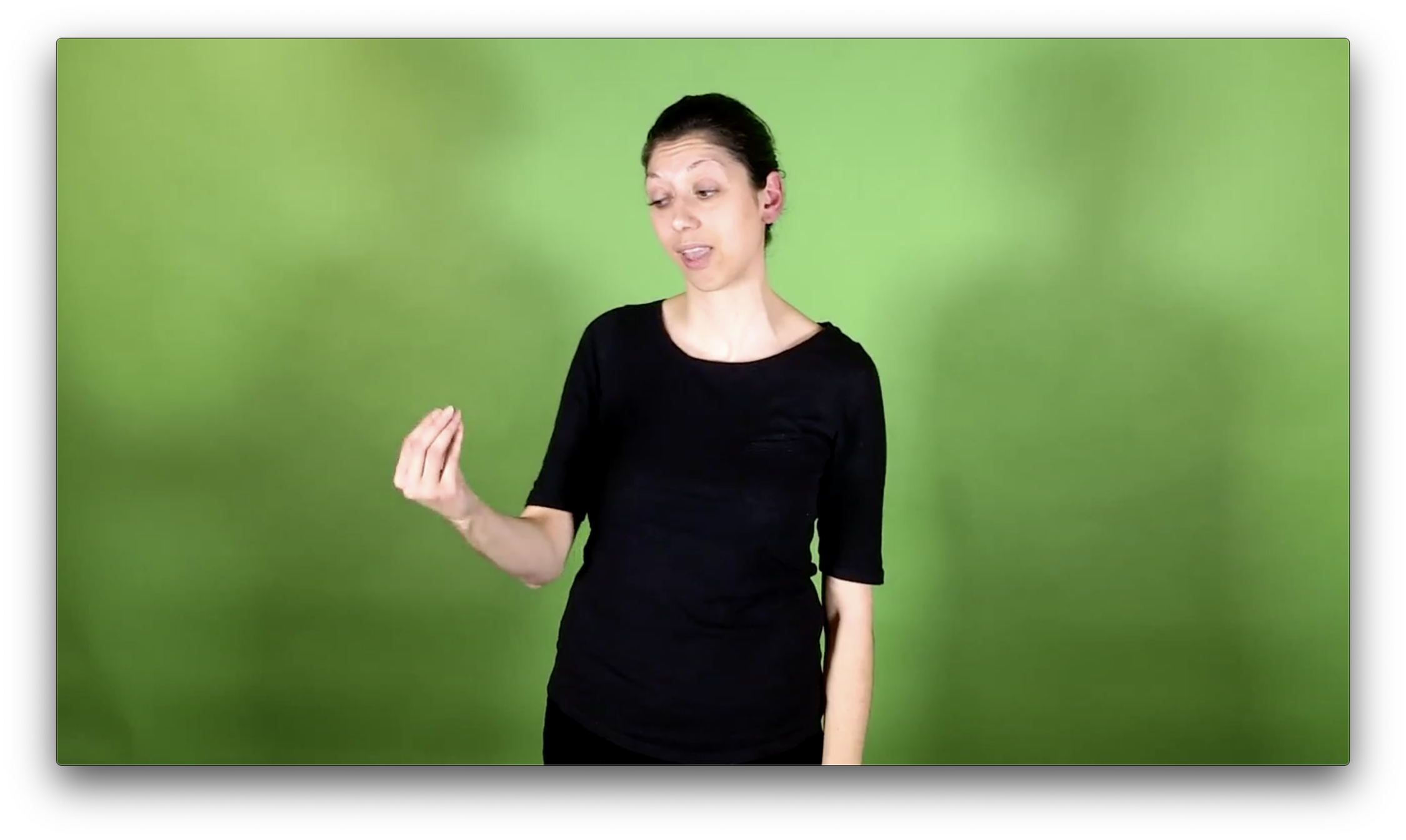

Supplement: S2 Fig — (TIF) [file pone.0236729.s007.tif]
