# Supplementary material for: Language experience in LSF development: Behavioral evidence from a sentence repetition task
Source: PLoS One. 2020 Nov 17;15(11):e0236729. doi: 10.1371/journal.pone.0236729 (PMC7671551; doi:10.1371/journal.pone.0236729)
Supplement: Attachment — Submitted filename: 2. PlosOne-Response-to-reviewers -R2.docx [file pone.0236729.s008.docx]

PONE-D-19-14457R1
Language experience in the phonological development of French Sign Language: Behavioural evidence from a sentence repetition task
PLOS ONE

Dear Mrs Bogliotti,

Thank you for submitting your manuscript to PLOS ONE. After careful consideration, we have decided that your manuscript does not meet our criteria for publication and must therefore be rejected.

Specifically:

==============================

Thank you for submitting your revised manuscript.  Two of the expert reviewers, who evaluated your original paper, and I have reviewed your work.

On the basis of these reviews (see below) and my own reading of the manuscript, I am afraid that I am unable to recommend your manuscript for publication.   Although we all agree that it is worthy and interesting area of research in the field, I hesitate to suggest that you attempt to undertake another revision. We appreciate the improvements made to the original manuscript, however, as you will see from the reviewers’ comments, a number of concerns expressed in the first round of reviews were not adequately addressed.

There remain considerable issues both from a conceptual and methodological perspective.

Comment 1# In particular, there has been a shift in focus on phonological development, however, as the reviewers point out, **the test measures selected may not be the most adequate to assess phonological skills**.

Response 1#. We agree that the SRT is not the most relevant test to assess phonological skills, and never we tried to convince the reader that it would be the case. However, we selected the SRT for the following reasons: 1) this task is known to assess general linguistic abilities in both spoken and sign languages; 2) moreover, it enables to point out quickly and reliably the linguistic level being altered. We would like to highlight that this task will be succeed only if phonological abilities are acquired.

We reformulated a part of the Introduction section in order to clarify this issue (pp 3 and pp 11).

Comment 2# Of importance is the lack of strong rationale for this new focus.

Response 2# We agree that we were not sufficiently clear to justify the rationale for the new focus in the R1. Importantly, we reorganized the whole Introduction section by focusing now on screening of general language abilities rather than only on phonological skills as it was the case in R1. We agree with previous commentaries of the Reviewers that pointed out that our initial focus on phonology was not really guided by an a priori hypothesis. Indeed, phonological difficulties in deaf children emerged from the SRT screening. We improved the manuscript by explaining that the results of the SRT in children allowed us to observe that most of the errors concerned the phonological level (see Results section).

Comment 3# The reviewers also highlight the need for a **clearer description of LSF** in the introduction rather than the general description of sign language provided.

Response 3# We are aware that a clearer description of the LSF would be relevant. However, we are willing to better introduce what is LSF for unfamiliar readers and to provide some references about general description of LSF. We added a new paragraph in the Introduction section pp 4 line 72-77.

Finally, there remain substantial concerns related to methodological issues and analyses.

Given the substantive nature and the extent of the concerns, I do not feel that we can consider the manuscript further for publication in PlOS One.

E. Fitzpatrick

Academic Editor

==============================

I am sorry that we cannot be more positive on this occasion, but hope that you appreciate the reasons for this decision.

Yours sincerely,

Elizabeth M. Fitzpatrick
Academic Editor
PLOS ONE

Additional Editor Comments (if provided):

I am writing regarding the review of your revised manuscript. Two of the expert reviewers, who evaluated your original paper, and I have reviewed your work. On the basis of these reviews (see below) and my own reading of the manuscript, I am afraid that I am unable to recommend your manuscript for publication. Although we all agree that it is worthy and interesting area of research in the field, I hesitate to suggest that you attempt to undertake another revision. We appreciate the improvements made to the original manuscript, however, as you will see from the reviewers’ comments, a number of concerns expressed in the first round of reviews were not adequately addressed. There remain considerable issues both from a conceptual and methodological perspective.

In particular, there has been a shift in focus on phonological development, however, as the reviewers point out, the test measures selected may not be the most adequate to assess phonological skills. Of importance is the lack of strong rationale/justification for this new focus. The reviewers also highlight the need for a clearer description of LSF in the introduction rather than the general description of sign language provided. Finally, there remain substantial concerns related to methodological issues and analyses.

Given the substantive nature and the extent of the concerns, I do not feel that we can consider the manuscript further for publication in PlOS One.

E. Fitzpatrick
Academic Editor


[Note: HTML markup is below. Please do not edit.]

Reviewers' comments:

Reviewer's Responses to Questions

**Comments to the Author**

1. If the authors have adequately addressed your comments raised in a previous round of review and you feel that this manuscript is now acceptable for publication, you may indicate that here to bypass the “Comments to the Author” section, enter your conflict of interest statement in the “Confidential to Editor” section, and submit your "Accept" recommendation.

Reviewer #1: (No Response)

Reviewer #3: (No Response)

2. Is the manuscript technically sound, and do the data support the conclusions?

The manuscript must describe a technically sound piece of scientific research with data that supports the conclusions. Experiments must have been conducted rigorously, with appropriate controls, replication, and sample sizes. The conclusions must be drawn appropriately based on the data presented.

Reviewer #1: Partly

Reviewer #3: Partly

3. Has the statistical analysis been performed appropriately and rigorously?

Reviewer #1: Yes

Reviewer #3: Yes

4. Have the authors made all data underlying the findings in their manuscript fully available?

The [PLOS Data policy](http://www.plosone.org/static/policies.action#sharing) requires authors to make all data underlying the findings described in their manuscript fully available without restriction, with rare exception (please refer to the Data Availability Statement in the manuscript PDF file). The data should be provided as part of the manuscript or its supporting information, or deposited to a public repository. For example, in addition to summary statistics, the data points behind means, medians and variance measures should be available. If there are restrictions on publicly sharing data—e.g. participant privacy or use of data from a third party—those must be specified.

Reviewer #1: Yes

Reviewer #3: Yes

5. Is the manuscript presented in an intelligible fashion and written in standard English?

PLOS ONE does not copyedit accepted manuscripts, so the language in submitted articles must be clear, correct, and unambiguous. Any typographical or grammatical errors should be corrected at revision, so please note any specific errors here.

Reviewer #1: Yes

Reviewer #3: No (we propose proofread the article by a native speaker of Englis for a second time).

6. Review Comments to the Author

Please use the space provided to explain your answers to the questions above. You may also include additional comments for the author, including concerns about dual publication, research ethics, or publication ethics. (Please upload your review as an attachment if it exceeds 20,000 characters)

Reviewer #1: Major Comments:

Comment4 # The revised manuscript has certainly improved, but still lacks focus. For instance, the reply letter and the introduction both briefly reference a debate over phonology in LSF, but the manuscript never explains what this is.

Response 4# We agree with this remark. We added a paragraph in which we present several references on phonological theory for SL and we suppressed the reference to the phonological debate (p. 26, line 572). Our position is that LSF can segmented in minimal units that can be called *phonemes*. In linguistics, a phoneme is a non-significant minimal unit which can be combined with other ones in order to create a meaningful linguistic sign. None definition propose that a phoneme is necessary a sound. Actually, the association of phoneme and sound is probably due to the long tradition of linguistic theories based on the studies of spoken languages.

Comment 5# The reader is left to guess that it might be either the very existence of phonology in LSF (which strikes this reviewer as bizarre) or the whether late-onset L1 acquisition is merely delayed or more deeply perturbed.

Response 5# Sorry, but we fail to understand this remark. Please could the reviewer precise what does she/he mean here? We will be happy to answer.

Comment 6# Nowhere do the authors explain how the design of their study will allow them to contribute to this debate, even though I think the data do in fact address the latter: the consistent lack of any CA x AoA interactions seems to imply that the performance on this sentence repetition task is merely delayed, rather than being more deeply perturbed.

Response 6# We understand what the reviewer means and we thank her/him to point out this issue. Nevertheless, we would like to indicate that our study, which is a transversal study, was not designed to disentangle between delay and deviance. The absence of a CA*AOA interaction suggests that the effect of AOA did not vary as a function of the CA. More important, it seems that the weight of AOA overpasses the effect of CA. This latter observation suggests that the delay observed in late signers cannot be compensated simply by cognitive development. Usually, the “more deeply disturbed” notion can be read in language disorder literature when one compares normo-typical participants *vs* participants with a language disorder. In this latter case, we know that the delay can never be caught because the cognitive structures are perturbed. In the present study, we cannot not conclude with respect to this issue. Further investigation will be necessary to disentangle between CA and AOA.

Comment 7 # (The authors make a passing mention to this debate in the discussion (p. 26 ln 586-588), but do not bother to say anything about which view their data support.) A third potential debate is introduced on p. 9 ln 191-199, but nothing is ever said about this again.

Response 7# In the general discussion, we suppressed the following sentence: “The systematic variation of AOA allowed us to better understand whether phonological development in late signers is only delayed or more deeply disturbed”. We replace it by the sentence: “The systematic variation of AOA allowed us to better understand to which extend sign language development is impacted by late acquisition” (p 24, lines 521-522). But in any case, it does not allow us to conclude in terms of language deviance. For this, it would be necessary to investigate the neural basis of language processing.

Comment 8 # There also remains the very puzzling question of whether this sentence repetition task is a valid source of insight about phonological development. There is not enough detail provided about the scoring system for me to evaluate whether this would in fact be a valid measure of LSF phonology.

Response 8# The scoring is presented in Table 2. We explained in details which phonological features (handshape, movement, location) were incorrectly reproduced as usual in the literature. In our view, that is the dependent variables allowing to evaluate LSF phonology.

Comment 9 #For example, it is unclear whether allophonic variations would be coded (e.g. “O” vs. “flat-O” handshape, or thumb adduction vs. abduction).

Response 9# The present study did not aim to investigate the phonetic variations of signs. The question of allophonic variation is very interesting but not relevant in the present study.

Comment 10# It is also unclear how signs would be categorized when they could be analyzed has belonging to more than one error category: for example, an error that yielded a minimal pair of the target sign (e.g. FATHER for MOTHER in ASL) could be analyzed as either a substituted sign or a phonological error. Decisions about how to score such trials could potentially have major consequences for the findings. If the paper is going to remain focused on phonology, more explicit details and their justification must be provided.

Response 10# We understand what reviewer means. However, in our LSF study with the linguistic material used, no such ambiguity was observed. However, if such errors would have been found, we would have scored as semantic substitution_other sign (see the scoring grid).

Comment 11 # It is hard to avoid the impression that the focus on phonological development is a rather post-hoc choice.

Response 11# We totally agree with Reveiewer#1 that by reading the Introduction section, readers can have the impression that phonology was not an *a priori* hypothesis. As above-mentioned, we first screened the global abilities using the SRT, and then we characterized the type of error (lexical, morphologic or phonological) and then based on the results we particularly focused on phonology as it was the less successful processing.

Comment 12# If this had been the goal a priori, there are many other paradigms that would have allowed more insight into phonological development than a sentence repetition task: for example, non-sign repetition, primed lexical decision, phonological similarity judgment, perceptual gating studies, and fluency tasks, just to name a few.

Response 12# We agree with Reviewer#1 that there is another paradigms and tasks in order to study phonological abilities. However, the goal of the present study was to provide scores on general language abilities and 1) to know the normal development in LSF; 2) to identify which level of language analysis was disturbed. As already explained, the SRT allowed us to test phonology as shown in previous clinical literature.

Comment 13# If the authors wish to maintain this focus, they will need to engage with the existing literature about native vs. non-native phonological development in other sign languages (e.g. ASL, BSL, NGT, DGS, inter al.), which is almost entirely absent from the present manuscript.

Response 13# We do not agree with this commentary. Indeed, we presented in different SL the literature of AOA for different processing levels (p 9 section “consequences on late SL acquisition”). Please note that in several studies, non-native signers refer to L2 hearing signers. In our study, non-native refers to delay of L1 acquisition.

Additional Comments:

Comment 14# Abstract: I find the use of AoA misleading here; it gives the impression that it was treated as a continuous variable, rather than a categorical one.

Response 14# We reformulated the Abstract in order to clarify this issue.

Comment 15# Introduction:
Despite the authors’ attempt to focus on LSF phonology, there are still many parts of the paper that are either irrelevant to or detracting from that focus. For example:
-p. 5 ln 103 through p. 6 ln 114.
-most of p. 9-14

Response 15# We agree and we reorganized the Introduction section by focusing more on the screening of general abilities and why we particularly focus the paper on phonology in a second step of the study.

Comment 16# The goal of providing benchmarks for the development of LSF phonology among older-L1 learners is reasonable. It’s just very different from what the authors have stated elsewhere. They need to make up their mind and figure out what their focus is.

Response 16# Our goal was not to precisely provide benchmarks for LSF phonological development. However, our data contribute to inform about language development in LSF. For avoiding confusions in the reader, we clarify the goal of the study.

Comment 17# p. 15 ln 343-344: Citations urgently needed regarding the two claims in this sentence: the impact of phonological skills on language ability (surely we all know people who are proficient L2 speakers despite having a strong L1 accent!), and the robustness of phonological development in predicting global linguistic skills in children.

Response 17# In our view, there is a misunderstanding of the sentence. What we wanted express here is that the phonological abilities in young children is a good predictor of future lexical knowledge, literacy, well-formed syntax etc. We were not referring to the supra-segmental characteristics observed in pronunciation of an L2. It is important to not confound phonetic and phonological abilities. We added a reference in order to clarify this issue.

Comment 18# Methods:
The adult participants should be described here.

Response 18# We agree and we added information about adult participants (lines 298-302)

Comment 19# No information on scoring reliability is provided.

Response 19# We agree and we added information about the two coders involved in the study. The inter-coder comparisons did not show reliable differences. And when there was some inter-coders variation then the first author of the study evaluated the production.

Comment 20# Results:
p. 21 ln 456: describe the factors and levels here.

Response 20# We added a description of the factors.

Comment 21# p. 22 ln 481-504: In my initial review, I expressed concern about the use of percentages as a metric when comparing groups that had denominators of different sizes. I think I did not express my concern adequately. In these sections, the authors report group differences in the prevalence of errors as a function of AoA and chronological age. They aim to convince the reader that these differences are in fact meaningful: in other words, **that there is something different about the way that these groups are processing the stimuli over and above their ability to recall the sentences**. That may indeed be the case, but the authors need to convince me that these differences are not merely epiphenomenal, due to the previously-reported differences in the number of signs recalled as a function of these same factors. Suppose two participants make a single “distortion” error (my term: meaning, an error on a sign that was recalled) on 5-sign sentence, but one participant recalled 5 signs while the other recalled 2 signs. That amounts to an “error” rate of 20% in the first participant and 50% in the second participant. But I would argue that we would be wrong to interpret that as a fact about the prevalence of distortion errors: it could simply be an epiphenomenon of overall accuracy. If I am to believe that there are in fact differences in the true rate of distortion errors, I would need to see additional analyses that take base recall rate into account: for example, a sub-analysis of utterances that were matched on the number of signs recalled, or that analyzed only trials with all items recalled correctly. I hope my concern is more clear this time around. Fortunately, this is not a problem in the Section 2 on phonological errors, where actual numbers are reported, rather than only percents.

Response 21# For each participant, we first calculated the percentage of produced sign. And then among these produced signs, we calculated the percentage of erroneous signs from the number of produced signs (ratio). These types of counting show the real difficulties of late signers. For example, they did a large number of phonological errors although they repeated less signs than the native signers.

Comment 22# p. 23 ln 513: The analysis described is a 2 x 3 ANOVA, not a 2 x 2.

Response 22# We corrected in 2 x 3 ANOVA.

Comment 23# p. 23 ln 515-517: The description of this analysis still gives the impression that Sign category and Phonological parameter were included as within-subject factors in the above 2 x 3 ANOVA, which is not in fact the case. This needs to be re-described to make it clear that these were separate analyses.

Response 23 # We corrected it.

Comment 24# Discussion:
p. 27 ln 611-612: This claim requires data that treat chronological age as a continuous variable, which was not provided. Furthermore, the failure to find differences between the older two groups further argues against this claim.

Response 24# We concluded p. 27 not on CA but on AOA. Anyway, it would not be possible to treat AOA as a continuous variable in our study as information given by the parents on this issue was not sufficiently precise (for example, “around 6 yrs.”)

Comment 25# p. 29 ln 668-669: “Contrary to expectations, we failed to observe a strong difference in phonological ability between native and late signers”. Either I have completely misunderstood this paper, or there is something wrong here. Perhaps the authors mean to say that they did not find differences among the two groups of older signers?

Response 25# We reformulated this sentence. Actually, we observed strong differences between AOA group. However, what we wanted to explain is that the nature of the errors at the qualitative level was not taken into consideration in this quantitative analysis.

Comment 26# p. 30 ln 689-691: I am additionally perplexed by the claims here, which seem both wrong and self-contradictory.

Response 26# We suppressed this sentence.

Comment 27# p. 30 ln 701-702: The authors must not criticize other researchers for lack of reliability without presenting reliability data of their own.
Response 27# As we made major changes in the paper, this sentence does no more appear in the revised paper (R2). However, we though reliability as “usuability”. It was not a criticism, but more an observation


Comment 28# Typographical Comments:
p. 5 ln 97: Begin a new paragraph at “Some studies have shown…”
p. 15 ln 356-358: fully redundant with information earlier in this paragraph
Table 1: The terms in this table need more explanation, particularly for the cases that appear to be exceptional somehow.
p. 24 ln 547: “AC” should be “CA”

Response 28# we made changes suggested by Reviewer#1

*******

Reviewer #3: Revision review:
In the revised manuscript, the authors addressed some of the key points raised in the review. I appreciate the improved clarity of the manuscript, and the narrowing of focus on phonological development in children acquiring LSF from varied backgrounds. An adult group of 10 native signers of LSF was added. These are the most significant improvements. I do have remaining concerns, some of which are carried over from the first review and some that have arisen in the revision, which I outline here:

CRationale:
Comment 29# I appreciate the clarification about how much information was available regarding age of exposure to LSF. If the goal is to separate the effects of chronological age and age of acquisition (AoA), then you need to control for a third, related, variable, which is years of experience with LSF: a 9 year old child who learned LSF at age 5 has had four years of experience; similarly a 4 year old child who learned LSF at birth has also had four years of experience, but it started at birth.

Response 29# We agree with the Reviewer#3 and we thank her/him for this commentary. Methodologically, it would be the procedure to follow. However, in practice, it is very difficult to obtain this information by the parents (and even from professionals) who don’t know only approximately the first contact with LSF.

Comment 30# I understand that precise AoA was not available among the late-learning group, and thus this relationship was not explored in this study, understandably because length of sign exposure was not available. However, what you end up with is that this study essentially compares deaf children with deaf vs. hearing parents.

Response 30# We agree, as it is the case in all studies comparing native vs. non native acquisition in SL.

Comment 31# This is in fact different from claiming that this is a sensitive test of AoA. AoA here is defined as anything other than birth (although some deaf children with hearing parents may have had very early exposure).

Response 31# We understand what the Reviewer means but, at least in France, such situation does not exist in deaf population. There is any SL nursery, and there are only three kindergarden with sign deaf teachers and elementary schools. These classes are frequented by native children. The current situation for deaf children from hearing parents is more an oralistic project, even if parents propose LSF courses to their child.

Comment 32# This is a different type of approach; albeit valid, but the authors need to be clear that they are **comparing two groups of children based on parental hearing status**.

Response 32# It is the intrinsic difference between native and late acquisition whatever the languages under investigation. L2 acquisition learners cannot have native parents in this L2.

Comment 33# It is also not accurate to say this is the first such study;

Response 33# In LSF, it is the first study. And in comparison, to the other SL, it is the first study with a large sample of participants (*N* = 64). For example, in the recent study of Rinaldi et al. (2018), only 23 children were tested.

Comment 34# as much other research has looked at deaf children as a function of parental hearing status, and not surprisingly found that deaf children with deaf parents tend to outperform those with hearing parents on measures of sign language ability. This may very well be the first such study in LSF, and this would be worth reporting.

Response 34# We thank the reviewer 3# for this fruitful remark and we tried to report this in the revised paper.

Comment 35 # The degree of explanation of basic features of sign language phonology and acquisition seems more appropriate for a review paper or textbook chapter. There is also a lack of **introduction to LSF**, a language with which most readers will be unfamiliar.

Response 35# We added a short description in the Introduction section and we cited the book of Millet (2019) (p. 4 line 72-77)

Comment 36 # I take the authors point that there is a lack of research on LSF phonology; nonetheless there have been several publications outlining features of LSF, and certainly **a brief historical introduction to the language** would be a valuable and necessary component of this paper. The authors cite some LSF research in the Discussion and there is a body of research on the language, so I am unclear as to why an Introduction to LSF is not possible.

Response 36# We understand this remark. As answered to Reviewer #1, we added in the revised version a short paragraph on description of LSF (p. 4 line 72-77)

Comment 37# • My suggestion regarding reducing the amount of general description was based on my general sense of the readership of PLOS ONE. Given the extent to which sign languages are now understood to be acquired and processed, this amount of information is extraneous and detracts from opportunities to describe the specific language (LSF) and the study. For example, when describing the effects of age of acquisition, given the current study’s focus on phonology, there is no need to discuss morphosyntactic delay in great detail. The authors could summarize this work in a sentence and maintain focus on the effects of AoA on phonological development (in fact there is much research on AoA effects on phonology in both L1 and L2 learners and this work is not mentioned here).

Response 37# We agree. As we made major changes in the paper, we think this point has been improved.

Comment 38# Similarly, the description of what phonology means in sign is likely to be known by most readers and feels too basic for this context. Finally, the full paragraph on handshape, movement, and location could be summarized more succinctly.

Response 38# Interestingly, Reviewer#1 has the exact opposite viewpoint which allows us to open debate. We tried to find a consensus for the naïve vs. expert reader.

This is especially true in light of the fact that the participants in this study are all school-aged and have had more than minimal exposure to LSF, and thus at least as far as initial acquisition have likely mastered the basic phonology of LSF.

Comment 39# Methods:
• If the analysis now focuses on phonological development, it is necessary to provide a rationale for a sentence repetition task, as well as a rationale for the ages of the participants. In fact in this study, sentences are essentially treated as a string of individual signs, because there is no analysis beyond the single sign level, and no consideration of the sentential context in which the signs appear.

Response 39# By definition, a sentence is failed when at least one word / sign is erroneous, and we observed errors in every sentence. None sentence was perfectly succeeded. Consequently, it was irrelevant to present this result.

Comment 40# Previous SRT tasks tend to look at sentence level scoring. Perhaps it would at least be worth looking at whether there were more errors in longer sentences vs. shorter sentences, or more errors in complex vs. simple sentences.

Response 40# It is a very good idea. However, as it was not the aim of the study to examine these two factors, we did not manipulate systematically them. Therefore, we are not able to conduct statistical analyses on these factors.

Comment 41# Figure 2 suggests that you have already coded sentences on at least two factors—number of signs, and complexity (four levels). It would be informative to know whether there was a higher overall repetition accuracy for shorter sentences, as one would expect; or if errors increased with sign complexity. **This type of information would lend some validity to the test as well, and I am not seeing a clear rationale for why none of these analyses are considered.**

Response 41# We are aware that this investigation might be important in a further step of the LSF SRT elaboration.

Comment 42# It would also be good to report the total number of signs—there are 20 sentences (but this unit of analysis is never used); how many individual signs were there in the dataset?

Response 42# The test was composed to 82 signs. The information is displayed in the Table 3 with the description of each sentences

Comment 43# Has the SRT been used with any sign languages with children in the current study’s age range? From a perspective of phonological development, most children have mastered basic phonology at this age; however the SRT task itself involves memory and syntactic processing and thus can be quite difficult at this age range.

Response 43# We are aware that the possible role of memory demands is a major criticism addressed to the SRT. For this reason, we discussed this issue in the initial version of the article. However, even we would like to assess the memory abilities in LSF, to date there is no standardized LSF tool available. Moreover, to succeed SRT it is necessary to have good general language abilities.

Thank you for defining SLI. I would avoid using phrases such as ‘Deaf SLI” or “SLI children” in favor of “(Deaf) children with SLI.”

Comment 44# Results:
• The authors acknowledge that age could and should be analyzed as a continuous variable (and in fact state that they did so), and yet all of the analyses maintain the three age group bands.

Response 44# Theoretically, it is possible to analyze AOA and CA as dimensional variables. However, in the present study we treated these variables as categorical ones.

Comment 45# Thank you for adding the adult data. Some overall information about adult performance in general would lend support for the task as appropriate for children. Also the goal of the adult group should be more explicit. What was predicted for adult performance, beyond the task being “not too hard nor too easy?”

Response 45# We predicted that adult should present high level of performances. This group was used to ensure that the LSF sentences were well constructed and an efficient test.

Comment 46# Do you have thoughts regarding how the adult performance might inform the children’s performance? For example, if there were particular signs or sentences that were difficult for native-signing adults (given that the task is designed for children ages 6 to 12), this may indicate something about the task more than the children’s abilities.

Response 46# It is a very interesting remark. As we consider that the adult group is the target model for children, we first expect that the native children perform as well as the adult. But to provide an appropriate answer, we need a qualitative analysis of errors. To date, we did not run yet qualitative analysis.

Comment 47# It looks like, as far as percent of signs repeated and percent errors, the 10-12 year old native signers and adults have similar patterns, which is suggestive that by 10 years, children have almost adult-like SRT performance. I would also add data on adult performance by error type (i.e. substitution, regionalism, etc.).

Response 47# these data are already presented in the figure 3 (new figure numbering). Concerning the similar pattern of performance, we think that the sentence repetition ability is relatively quickly mastered in children (around 8-9 years), but the difference of performance is observed in the number of phonological errors)

Comment 48# Percentage of incorrect sign repetition according to sign category: It seems that perhaps there were no effects here because the vast majority of errors were in the sign variant (phonological error) category rather than the substitution or regionalism category; if so that could be discussed.

Response 48# We found a little effect, undoubtedly due to the “regionalism” substitutions. And the results regarding the sign variant are discussed in the phonological paragraph in the Discussion section (since p 26 line 563).

Comment49# • I’m still confused about the ANOVA described here: A 2 x 2 ANOVA with AOA (2 levels: native signers and late signers) and CA (3 levels: 6-7, 8-9 and 10-12 years-old) considered as between-subject factors was run on the number of phonological errors. Sign category (3 levels: Frozen Sign, Dominant-Hand Classifier and Non-Dominant-Hand Classifier) and Phonological parameters (3 levels: Handshape, Movement, Location) were considered as within-subject factors. Were these all put into a single ANOVA? Or was it first an ANOVA on total phonological errors (by age group and AoA group), and then an ANOVA on Sign Category errors (by age group and AoA group) and then an ANOVA on phonological parameters?

Response 49# Our analysis corresponded to the last proposal (an ANOVA on total phonological errors (by age group and AoA group), and then an ANOVA on Sign Category errors (by age group and AoA group) and then an ANOVA on phonological parameters). We tried to disambiguate this point in the Result section.

Comment 50# I am unclear what the percentages in Figure 7 are indicating. The shaded areas show a percentage of phonological errors of each parameter as a function of what?

Response 50# It is an error ratio. We first analyse the number of phonological errors (figure 4), then we look the proportion of phonological parameter errors. So native signers made significantly less errors than late signers, but present the same proportion of phonological errors (figure 6) : handshape and movement is more difficult to mastered than location. In adults, less errors than children and if they fail a parameter, it is more often the handsphape (as we found in the literature) because this parameter is a fine grained parameter and need to be more accurate in perception and production

Comment 51# Discussion:
• If the goal is to determine whether phonological development is delayed or deficient in late signers, how do differences in CA and AoA help inform this question?

• The discussion contains some data about scoring the quality of the repetition at the sentence level regardless of the errors; I’d suggest including this data in the Results.

• The discussion about the SRT not being appropriate for measuring morpho-syntax I believe is a reflection of the approach to analysis (i.e. analyzing phonology at the single sign level only).

Response 51# we made major changes in the Discussion section and reorganized the different paragraphs. The rewiewer #3 will find the responses in the Discussion of the revised version.

7. PLOS authors have the option to publish the peer review history of their article ([what does this mean?](https://journals.plos.org/plosone/s/editorial-and-peer-review-process#loc-peer-review-history)). If published, this will include your full peer review and any attached files.


If you choose “no”, your identity will remain anonymous but your review may still be made public.


**Do you want your identity to be public for this peer review?** For information about this choice, including consent withdrawal, please see our [Privacy Policy](https://www.plos.org/privacy-policy).

Reviewer #1: No

Reviewer #3: No


[NOTE: If reviewer comments were submitted as an attachment file, they will be attached to this email and accessible via the submission site. Please log into your account, locate the manuscript record, and check for the action link "View Attachments". If this link does not appear, there are no attachment files to be viewed.]

- - - - -
For journal use only: PONEDEC3
